# Dynamics of gamete production and mating in the parasitic protist *Trypanosoma brucei*

**DOI:** 10.1186/s13071-016-1689-9

**Published:** 2016-07-20

**Authors:** Lori Peacock, Mick Bailey, Wendy Gibson

**Affiliations:** School of Biological Sciences, University of Bristol, Bristol, BS8 1TQ UK; School of Clinical Veterinary Science, University of Bristol, Langford, Bristol, BS40 7DU UK

**Keywords:** *Trypanosoma*, Tsetse, Mating, Sexual reproduction, Hybrids, Sequential infection, Immune response, Competition, Human infectivity

## Abstract

**Background:**

Sexual reproduction in *Plasmodium falciparum* and *Trypanosoma brucei* occurs in the insect vector and is important in generating hybrid strains with different combinations of parental characteristics. Production of hybrid parasite genotypes depends on the likelihood of co-infection of the vector with multiple strains. In mosquitoes, existing infection with *Plasmodium* facilitates the establishment of a second infection, although the asynchronicity of gamete production subsequently prevents mating. In the trypanosome/tsetse system, flies become increasingly refractory to infection as they age, so the likelihood of a fly acquiring a second infection also decreases. This effectively restricts opportunities for trypanosome mating to co-infections picked up by the fly on its first feed, unless an existing infection increases the chance of successful second infection as in the *Plasmodium*/mosquito system.

**Results:**

Using green and red fluorescent trypanosomes, we compared the rates of trypanosome infection and hybrid production in flies co-infected on the first feed, co-infected on a subsequent feed 18 days after emergence, or fed sequentially with each trypanosome clone 18 days apart. Infection rates were highest in the midguts and salivary glands (SG) of flies that received both trypanosome clones in their first feed, and were halved when the infected feed was delayed to day 18. In flies fed the two trypanosome clones sequentially, the second clone often failed to establish a midgut infection and consequently was not present in the SG. Nevertheless, hybrids were recovered from all three groups of infected flies. Meiotic stages and gametes were produced continuously from day 11 to 42 after the infective feed, and in sequentially infected flies, the co-occurrence of gametes led to hybrid formation.

**Conclusions:**

We found that a second trypanosome strain can establish infection in the tsetse SG 18 days after the first infected feed, with co-mingling of gametes and production of trypanosome hybrids. Establishment of the second strain was severely compromised by the strong immune response of the fly to the existing infection. Although sequential infection provides an opportunity for trypanosome mating, the easiest way for a tsetse fly to acquire a mixed infection is by feeding on a co-infected host.

## Background

In tropical Africa the protozoan parasite *Trypanosoma brucei* causes sleeping sickness (Human African Trypanosomiasis) and is one of the causative organisms of the livestock disease, nagana, along with other tsetse-transmitted trypanosome species such as *T. congolense* and *T. vivax*. Sexual reproduction involving a meiotic division and production of haploid gametes has been demonstrated in *T. brucei* during its development inside the salivary glands (SG) of experimentally infected tsetse flies [1, 2]. Production of trypanosome hybrids only occurs within SG that contain a mixture of different *T. brucei* strains, and thus the key requisite for mating is that a tsetse fly becomes infected with more than one trypanosome strain. It has been assumed that a fly is most likely to acquire a mixed infection on its first bloodmeal after emergence from the puparium, as this is the peak of susceptibility and flies become refractory to infection as they age [3–8]. The bloodmeal triggers physiological changes in the fly including extrusion of the peritrophic matrix that holds the bloodmeal and prevents interaction of pathogens with the gut epithelium [9, 10] and up-regulation of innate immune defences such as secretion of lectins and anti-microbial peptides [8, 10–14]. These observations go some way to explain the very low trypanosome infection rates recorded for wild-caught flies, typically < 0.1% of tsetse have SG infected with *T. brucei* ([15] and examples reviewed therein).

These barriers to fly infection restrict the likelihood of more than one trypanosome strain reaching the fly SG and having the opportunity to mate, with the consequence that trypanosome mating in nature may be extremely rare. However, under certain conditions, for example starvation, later trypanosome infections can establish in the SG [16–18], opening the possibility that sequential infection may also lead to mixed infection and the production of trypanosome hybrids. The presence of an existing infection may even enhance establishment of a second infection, as recently shown in the mosquito-*Plasmodium* system [19]. The possibility of sequential infection would have implications for the frequency of mating between the human pathogen *T. b. rhodesiense* (*Tbr*) and the non-human-infective *T. b. brucei* (*Tbb*), as both subspecies cannot co-occur in humans. A fly could become co-infected with *Tbr* and *Tbb* only by feeding on an infected reservoir host with a mixed infection, unless sequential infection resulting from separate feeds on different hosts were possible.

It is known that there are strain differences in the time taken for *T. brucei* to complete its full developmental cycle from the initial infective bloodmeal to production of SG metacyclics, with the fastest maturing strains producing infective metacyclics in about 2 weeks [20]. SG colonization is accomplished by migration of trypanosomes from the established midgut population, but it is not clear whether invasion happens only once with a single wave of migratory forms, or if it is continuous over the life of the fly [21, 22]. For sexual reproduction to occur in *T. brucei*, the developmental pathways of two or more mating-compatible strains need to intersect such that gamete production in the SG is synchronous. Hybrids have been detected as early as 13 days after infection [23], suggesting that mating occurs as soon as trypanosomes invade the SG, and hybrid production has been observed throughout the duration of SG infection [24]. It follows that production of meiotic and gamete stages is not limited to a particular phase of SG infection, but results to date indicate that the number of meiotic trypanosomes peaks around 20 days after the infective bloodmeal [1, 2], suggesting similar timing for peak gamete production. This would lead to a restricted window of opportunity for trypanosome mating.

Here we compared the outcome of genetic crosses in which tsetse flies had been infected with both parental trypanosomes on the first blood feed as tenerals, or co-infected on a subsequent feed 18 days after emergence (non-teneral), or fed sequentially with each trypanosome clone 18 days apart (sequential). We also investigated the timecourse of production of migratory forms, meiotic dividers and gametes in samples of salivary exudate from individual flies that were sequentially infected with two different trypanosome strains.

## Methods

### Trypanosomes

*T. b. brucei* clones 1738G (MOVS/KE/70/1738) transfected with a gene for enhanced green fluorescent protein (GFP), and J10R (MCRO/ZM/73/J10 CLONE 1) transfected with a gene for modified red fluorescent protein (RFP), were crossed in tsetse, producing the red and green fluorescent F1 hybrid clones F1R1 and F1G2, respectively [23]. These four clones were used in the experiments reported here, together with J10G, transfected with a gene for enhanced GFP. These trypanosome clones were shown to be mating compatible in previous crosses [23, 25]. Trypanosomes were grown as bloodstream forms in mice or as procyclics in Cunningham’s medium (CM) at 27°C.

### Tsetse infections

**Fig. 1:**
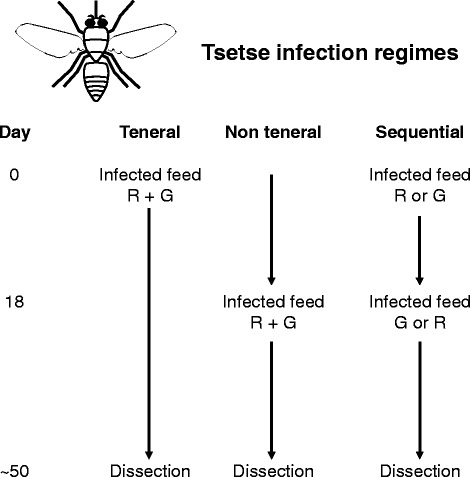
Experimental design. Tsetse flies were fed red (R) and green (G) fluorescent *T. b. brucei* clones in three different regimes: Teneral, together in the first bloodmeal as newly-emerged flies; Non-teneral, together on day 18; or Sequential, R or G on day 0, followed by the other colour on day 18. Flies were assigned to each treatment from the same batch of emergents and all flies received an infected or uninfected bloodmeal on the same day (day 0); non-teneral and sequential groups also received an infected bloodmeal on day 18; otherwise, flies were fed sterile horse blood (black arrows) until dissection

Tsetse flies (*Glossina morsitans morsitans* or *G. pallidipes*) were caged individually or in groups of 15–25. There is no difference in the developmental cycle of *T. brucei* in these two flies as far as we are aware, but SG invasion and colonisation is more efficient in *G. pallidipes* [26], making it easier to obtain large numbers of flies with SG infection. Flies were kept at 25 °C and 70 % relative humidity, and fed on sterile defibrinated horse blood via a silicone membrane. Male flies were given an infected bloodmeal either at their first feed 1–2 days after emergence (teneral), or at a subsequent feed 18 days after the first feed (non-teneral); sequentially infected flies received infected bloodmeals at both timepoints (Fig. 1). Non-teneral flies were starved for 4–7 days before the 18 day infective feed to increase infection rates [16, 18]. We found that flies starved 7 days before infection with F1R1 had a significantly higher midgut infection rate (26/41 = 63 %) than did flies starved 4 days before infection with F1R1 (10/41 = 24 %) (Fisher’s exact test: *P* = 0.0007). While 7 day starved flies had a higher transmission index (5/26 = 19 %) than did 4 day starved flies (1/10 = 10 %), this was not significant. Infective feeds consisted of cryopreserved bloodstream form trypanosomes (approximately 10^6^ cells/ml) in defibrinated horse blood, or procyclic trypanosomes (approximately 10^7^ cells/ml) in washed horse red blood cells resuspended in Hank’s Balanced Salt Solution. Infective bloodmeals were supplemented with 10 mM L-glutathione to increase infection rates [27]. We tested whether glutathione would also boost midgut infection rates of non-teneral flies given the infected bloodmeal 18 days after emergence. Although small compared to the > 80 % midgut infection rate achieved with teneral flies, there was a significant increase in the midgut infection rate for non-tenerals: 8/44 (9 %) infection rate with glutathione versus control flies 0/39 (0 %) (Fisher’s exact test: *P* = 0.006).

### Salivary probes

Individually caged flies were allowed to probe warm microscope slides 11–18 days after the first infected bloodmeal as described [28]. This procedure was repeated on days 29–42, i.e. 11–24 days after the second infected bloodmeal. Cells were fixed in 2 % w/v paraformaldehyde (PFA) at room temperature for 20 min, stained with DAPI in VECTASHIELD mounting medium (Vector Laboratories) to visualize the nucleus and kinetoplast and viewed by fluorescence microscopy using a DMRB microscope (Leica) equipped with a Retiga Exi camera (QImaging) and Volocity software (PerkinElmer). Digital images of life cycle stages were analysed using Image J (https://imagej.nih.gov/ij/). Morphology and relative positions of the nucleus and kinetoplast were used to identify developmental stages [1, 2, 22]. Cells were assigned to the following developmental stages: long proventricular trypomastigote, asymmetrically dividing cell (including short and long epimastigotes), epimastigote, metacyclic, meiotic dividing epimastigote, gamete.

### Dissection

Individually caged flies were dissected 36 days after the second infected feed on day 18 (= day 54); other flies were dissected up to 32 days following the second infective feed (= day 50). Fly organs (salivary glands and alimentary tract from the proventriculus to the hindgut) were dissected in separate drops of phosphate buffered saline (PBS) and examined for the presence of fluorescent trypanosomes. Metacyclics from infected salivary glands were inoculated into mice; bloodstream forms were subsequently transformed back to procyclics by incubation in CM at 27 °C. For trypanosome counts, tsetse alimentary tracts were dissected in a drop of PBS, placed in 50 μl PBS in a microcentrifuge tube and thoroughly disrupted using a Teflon pestle. The trypanosomes were fixed by adding 0.1 % w/v PFA in PBS and counted under fluorescence using a haemocytometer. Isolation and analysis of hybrid progeny were as described previously [23, 29] using seven microsatellite loci for genotyping [30].

### Statistical analysis

Fisher’s exact test was used for analysis of infection rates using http://www.graphpad.com/quickcalcs/contingency1/. Numbers of trypanosomes were log transformed prior to analysis to normalise variances. ANOVA and correlation data were processed using the statistical package SPSS 23.

## Results

### Midgut infection rates in non-teneral flies

We compared infection rates of two distinguishable *T. b. brucei* strains (green fluorescent F1G2 and red fluorescent F1R1) in different infection regimes: 1) Teneral, co-infection of newly-emerged flies with F1G2 and F1R1 on their first feed; 2) non-teneral, co-infection of 18 day old flies; 3) and 4) sequential, newly-emerged flies fed with F1G2 or FIR1, followed by an infected bloodmeal with the other strain on day 18 (Fig. 1). Flies were dissected on days 49–50 and the results are shown in Table 1. All teneral flies were infected and had both trypanosome clones in their midguts (infection regime 1). As expected, the midgut infection rate was greatly reduced in the non-teneral flies (infection regime 2; 49 % compared to 100 %, Fisher’s exact test: *P* = 0.0001), but 83 % of the infected midguts contained both clones (15/18). For the sequentially fed flies (infection regimes 3 and 4), although the midgut infection rates were high, most infections were of the trypanosome clone fed on the first infected feed to teneral flies; combining data from both sequential regimes, only 7 % (5 of 69) of midguts had both clones. Compared to the non-teneral flies, a significantly lower percentage of the sequentially-fed flies became infected from the day 18 bloodmeal [49 % (18/37) compared to 9 % (7/75); Fisher’s exact test: *P* = 0.0001]. Thus, establishment of the second infected clone is strongly negatively influenced by the presence of an existing midgut infection.

**Table 1:** Trypanosome infection rates in teneral and non-teneral flies. Flies (*Glossina morsitans morsitans*) were fed two *T. b. brucei* clones F1G2 and F1R1 (1) together on their first bloodmeal as tenerals (newly-emerged flies), or (2) together on day 18 as non-tenerals, or (3) and (4) sequentially 18 days apart, as shown in Fig. 1. Midguts and salivary glands (SG) were scored for the presence of each clone by colour of fluorescence. Asterisks indicate that yellow hybrid trypanosomes were observed in these SG infections. TI is the transmission index (percentage of infected midguts that established SG infection)

Infection regime	Midguts				Salivary glands			
	Infection rate	Trypanosomes present			TI	Trypanosomes present		
		Both	F1G2	F1R1		Both	F1G2	F1R1
1 Teneral F1G2 + F1R1 on day 0	100 % (28/28)	100 % (28/28)	0 % (0/28)	0 % (0/28)	32 % (9/28)	44 % (4/9)*	56 % (5/9)	0 % (0/9)
2 Non-teneral F1G2 + F1R1 on day 18	49 % (18/37)	83 % (15/18)	6 % (1/18)	11 % (2/18)	22 % (4/18)	25 % (1/4)*	75 % (3/4)	0 % (0/4)
3 Sequential F1G2 on day 0, F1R1 on day 18	89 % (34/38)	6 % (2/34)	91 % (31/34)	3 % (1/34)	47 % (16/34)	0 % (0/16)	100 % (16/16)	0 % (0/16)
4 Sequential F1R1 on day 0, F1G2 on day 18	95 % (35/37)	9 % (3/35)	3 % (1/35)	89 % (31/35)	20 % (7/35)	0 % (0/7)	14 % (1/7)	86 % (6/7)

This result was confirmed in a second experiment where F1R1 was fed on day 18 either alone, or after an initial feed with F1G2, or parental clones, 1738G and J10G (Table 2). F1R1 established significantly more midgut infections in flies that had received no prior infective feed (infection regime 1) compared to flies that had been fed an infective feed as tenerals (infection regimes 2 - 4) (Fisher’s exact test: *P* = 0.002), irrespective of which trypanosome clone was used for the first infected feed. These clones vary in their ability to establish infection and in previous experiments we showed that F1 clones could outcompete their parents as procyclics in the fly midgut when co-infected in teneral flies [25]. We conclude that competition plays a minor role, if any, in the failure of the second trypanosome clone to establish in sequentially fed flies.

**Table 2:** Trypanosome infection rates in sequential feeds. Flies (*Glossina morsitans morsitans*) were fed parental (1738G, J10G) or F1 trypanosome clones (F1R1, F1G2) as tenerals or non-tenerals on day 18 according to infection regimes 1–4 and dissected 24–25 days later. Midguts and salivary glands (SG) were scored for the presence of each clone by colour of fluorescence

Infection regime			Midguts				Salivary glands			
	Teneral feed	18 day feed	Infection rate	Trypanosomes present			TI	Trypanosomes present		
				Both	R only	G only		Both	R only	G only
1	–	F1R1	42 % (8/19)	na	100 % (8/8)	na	0 % (0/8)	na	0 % (0/0)	na
2	1738G	F1R1	83 % (10/12)	20 % (2/10)	0 % (0/10)	80 % (8/10)	20 % (2/10)	50 % (1/2)*	0 % (0/2)	50 % (1/2)
3	J10G	F1R1	86 % (12/14)	0 % (0/12)	17 % (2/12)	83 % (10/12)	8 % (1/12)	0 % (0/1)	0 % (0/1)	100 % (1/1)
4	F1G2	F1R1	85 % (11/13)	9 % (1/11)	9 % (1/11)	82 % (9/11)	9 % (1/11)	0 % (0/1)	0 % (0/1)	100 % (1/1)

Asterisk indicates that yellow hybrid trypanosomes were observed in this SG infection. TI is the transmission index (percentage of infected midguts that established SG infection). *Abbreviation*: na, not applicable

**Fig. 2:**
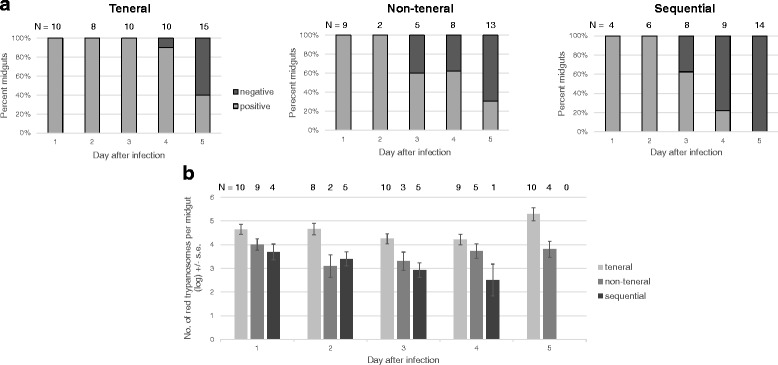
Timecourse of initial establishment (days 1–5) of midgut infection in teneral, non-teneral and sequentially-fed flies. Flies (*Glossina morsitans morsitans*) were fed on a bloodmeal containing F1R1 and F1G2 as tenerals, 18 day non-tenerals, or sequentially with the F1R1 infective feed 18 days after the first feed with F1G2. **a** Percent of midguts that were positive or negative for FIR1 trypanosomes upon dissection; all midguts were positive for F1G2. The sequentially fed flies had significantly more negative midguts than both teneral (Fisher’s exact test: *P* = 0.02) and non-teneral flies (Fisher’s exact test: *P* = 0.04) at day 5. *N* = number of flies. **b** Average counts (log ± se) of F1R1 trypanosomes per macerated positive midgut. Treatment had a significant effect on the number of trypanosomes in midguts (ANOVA: *F*_(2,77)_ = 25.15, *P* < 0.0001), with significantly lower numbers of trypanosomes in the positive midguts for the sequentially fed flies than either teneral (*Post-hoc* LSD test: *P* < 0.0001) or non-teneral flies (*Post-hoc* LSD test: *P* = 0.04), and significantly lower numbers for non-teneral than teneral flies (*Post-hoc* LSD test: *P* < 0.0001). N = number of flies; trypanosome negative flies have been excluded from the total

To investigate what happens to the second trypanosome, we compared the numbers of trypanosomes in midguts of teneral and non-teneral flies with or without an existing trypanosome infection on days 1-5 post-infected feed (Fig. 2); this is the critical time window for midgut infection to establish [31]. While the percentage of trypanosome-negative midguts increased for all feeding regimes over the timecourse (Fig. 2a), the sequentially fed flies with an established infection of F1G1, showed very poor establishment of the second fed trypanosome, F1R1, with significantly more negative midguts than both teneral (Fisher’s exact test: *P* = 0.02) and non-teneral flies (Fisher’s exact test: *P* = 0.04) at day 5, although all flies were fed exactly the same infective bloodmeal. In addition, treatment had a significant effect on the number of trypanosomes in midguts (ANOVA: *F*_(2,77)_ = 25.15, *P* < 0.0001), with significantly lower numbers of trypanosomes in the positive midguts for the sequentially fed flies than either teneral (*Post-hoc* LSD test: *P* < 0.0001) or non-teneral flies (*Post-hoc* LSD test: *P* = 0.04), and significantly lower numbers for non-teneral than teneral flies (*Post-hoc* LSD test: *P* < 0.0001) (Fig. 2b). Thus, although the fly midgut environment is clearly more hostile to trypanosome infection at day 18 than day 0, there is an additional negative impact of a pre-existing trypanosome infection. Possible explanations are a specific anti-trypanosome immune response raised by the fly to the first-fed trypanosomes, or direct competition for resources when there is a pre-existing trypanosome infection. It is unlikely to be competition, because there was a significant positive correlation between the counts of red and green trypanosome clones in each sequentially fed fly midgut (Correlation: *r*_(35)_ = 0.83, *P* = 0.03), instead suggesting variation in fly susceptibility to infection.

### Salivary gland infections in non-teneral flies

Invasion and colonisation of the salivary glands (SG) is a major bottleneck during the trypanosome life cycle; many midgut infections fail to establish SG infection at all, and successful infections are founded by the relatively small numbers of trypanosomes that reach the SG. Here, less than half the midgut infections resulted in SG infections, with no significant difference in the overall transmission index (TI; percentage of infected midguts that establish SG infection) between the teneral and non-teneral flies that were either co-infected or sequentially infected with F1G2 and F1R1 (Table 1). A significantly lower percentage of the sequentially fed flies (infection regimes 3 and 4) had SG infected with the trypanosome clone infected on day 18 (combined TI of 1/69 = 1.5 %) than did non-teneral flies (TI of 4/18 = 22 %) (Fisher’s exact test: *P* = 0.006). Five SG in infection regimes 1 and 2 were colonised by both F1G2 and F1R1, and yellow hybrid trypanosomes were also produced in some of these co-infected SG (Table 1, asterisks), notably in the mixed SG of the non-teneral flies fed both trypanosomes at day 18. SG infection rates in the second set of sequential feeding experiments were low overall and the second trypanosome (F1R1) rarely established an infection (Table 2). Despite this very low success, the sequential infection of F1R1 after 1738G resulted in one SG with a mixed infection and yellow hybrid trypanosomes (Table 2, asterisk).

### Sexual reproduction in non-teneral flies

We repeated this successful cross to examine the frequency of hybrid trypanosome production in two transmission regimes: (1) infecting non-teneral flies with a bloodmeal containing a mixture of 1738G and F1R1 (referred to as ntGR); (2) infecting teneral flies with 1738G followed 18 days later with F1R1 (referred to as GR). The results are shown in Table 3. For cross ntGR, over half the flies (53 %) had a midgut infection upon dissection and a transmission index of 90 %. The majority of infected midguts examined had both trypanosomes present. We have previously shown that the composition of trypanosome clones can differ between the glands of an individual fly [23], and therefore examined the trypanosome population of each SG individually. Of the infected SG, 41 % contained both trypanosome clones and of these 75 % had yellow hybrid trypanosomes present, showing that not only did mating occur, but it happened at a high frequency.

**Table 3:** Hybrid production in non-teneral flies. Summary of midgut and salivary gland (SG) infection results (*Glossina pallidipes*) when trypanosome clones 1738G and F1R1 were either co-transmitted in non-teneral flies (ntGR) or sequentially, with 1738G fed to teneral flies and F1R1 fed to these flies 18 days later (GR). Midguts from a subsample of flies and all SGs were scored for the presence of one or both trypanosome clones; additionally, each SG was examined for yellow hybrids. TI is the transmission index, which is the percentage of infected midguts that established SG infections

Cross	Midguts				Salivary glands				
	Infection rate	Trypanosomes present			TI	Trypanosomes present per SG			
		Both	R only	G only		Both	R only	G only	Yellow (% of R&G)
ntGR	39/74 (53 %)	20/25 (80 %)	4/25 (16 %)	1/25 (4 %)	35/39 (90 %)	28/68 (41 %)	31/68 (46 %)	9/68 (13 %)	21/28 (75 %)
GR	60/66 (91 %)	20/44 (46 %)	8/44 (18 %)	16/44 (36 %)	53/60 (80 %)	21/101 (21 %)	17/101 (17 %)	63/101 (62 %)	13/21 (62 %)

For the sequential cross GR, the midgut infection rate was very high at 91 %, with a transmission index of 80%. Less than half (46 %) of the infected midguts examined contained both trypanosome clones, which was significantly lower than for cross ntGR (80 %) (Fisher’s exact test: *P* = 0.006), with over a third (36 %) having only 1738G, the clone in the first infected bloodmeal. As a consequence, a significantly lower number of SG contained a mixed population (21 %) than did SG from the ntGR cross (41 %) (Fisher’s exact test: *P* = 0.006), but of these GR SG, 62 % contained yellow hybrid trypanosomes in their glands, showing that mating had occurred at high frequency. The production of hybrid trypanosomes in crosses ntGR and GR was verified by cloning and genotyping the progeny clones using seven microsatellite loci. Three hybrid genotypes were recovered from each cross (Table 4).

**Table 4:**
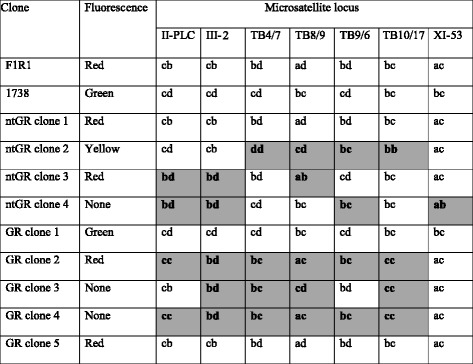
Summary of genotype data for parental and progeny clones. Cross ntGR: non-tenerals 1738G x F1R1. Cross GR: 1738G followed by F1R1 18 days later. Shaded cells indicate shared parental inheritance of alleles in hybrid clones

In summary, both transmission regimes allowed mixed infections to establish in the midguts and SG of infected flies and led to the production of hybrids. However, it can be seen from Table 3 that the dynamics of invasion of the SG tipped in favour of the first fed trypanosome 1738G in cross GR. Whereas the relative proportion of SG with 1738G and/or F1R1 was 37:59 in the ntGR cross, it changed to 84:38 in the GR cross (Fisher’s exact test: *P* = 0.0001), where 1738G was fed first. We therefore examined the relative timing of invasion of the SG, developmental stages and production of gametes of each trypanosome clone in sequentially infected flies.

### Developmental and sexual stages in sequentially infected flies

**Fig. 3:**
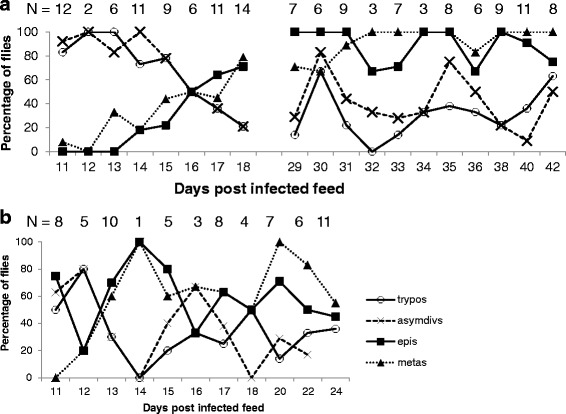
Trypanosome developmental stages from salivary exudate. Trypanosome developmental stages recorded from salivary exudates from flies (*Glossina pallidipes*) infected with **a** 1738G and **b** F1R1. Percentage of flies with trypanosomes in exudates that contained the development stage; *n* = number of flies with a sample containing trypanosomes on that day. Flies were given an infective feed containing 1738G on day 0, and subsequently fed a further infective feed containing F1R1 on day 18; the experiment continued to day 42. From day 11 onwards, flies were encouraged to probe on warmed glass slides before feeding. Results were initially obtained for 1738G alone (timecourse A, days 11–18), and then for both 1738G and F1R1, starting 11 days after the second infected feed with F1R1 (1738G, timecourse A, days 29–42; F1R1, timecourse B, days 11–24). *Abbreviations*: *trypos* proventricular trypomastigotes, *asymdiv* asymmetric dividers, *epis* salivary gland epimastigotes, *metas* metacyclics

**Fig. 4:**
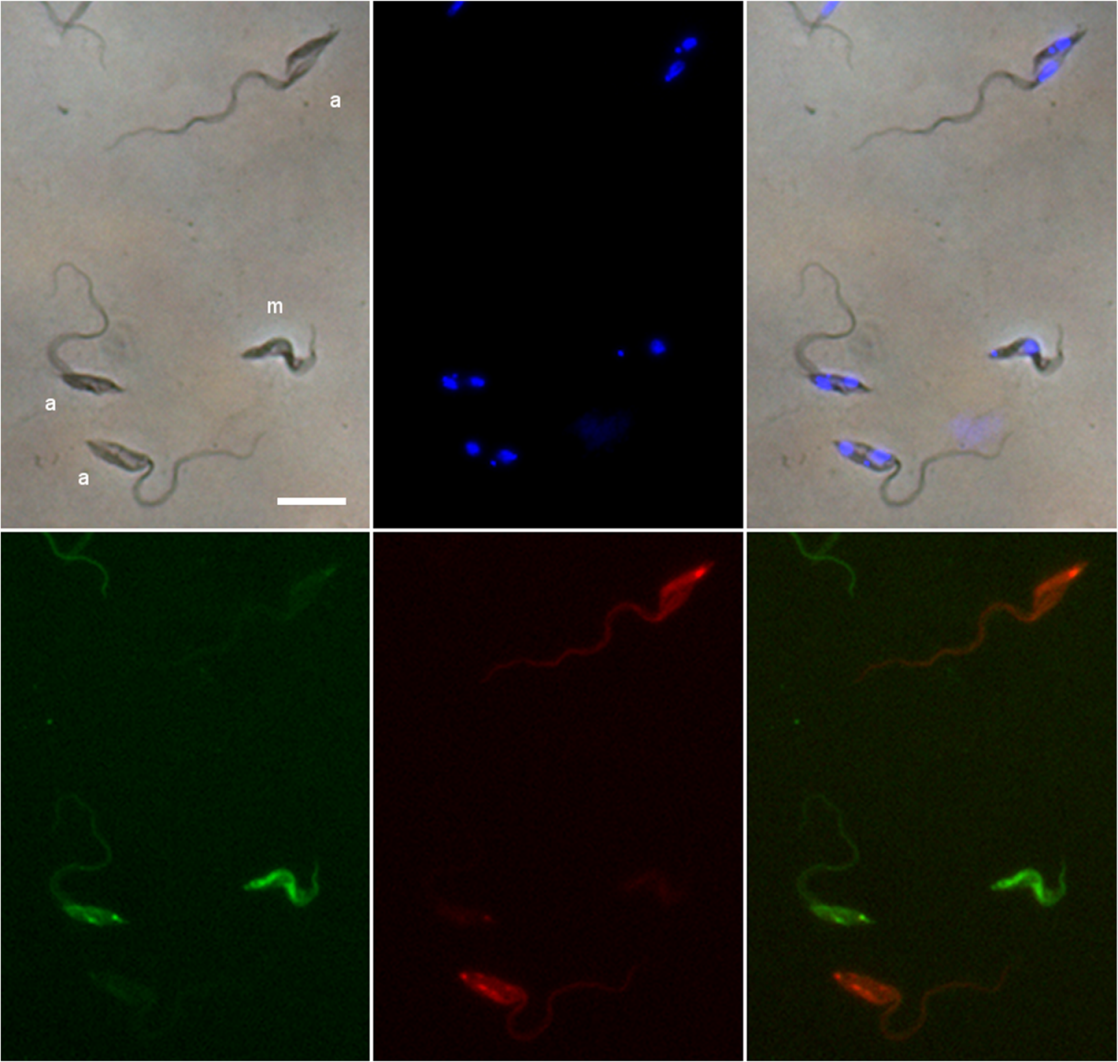
Trypanosome migratory and developmental stages in salivary exudate. Trypanosomes in the salivary probe of a sequentially infected fly (*Glossina pallidipes*) 35 days after infection with 1738G and 17 days after infection with F1R1. Asymmetric dividers (a) of both trypanosome clones are present as well as metacyclics (m). From left to right, upper row, brightfield, DAPI and merge; lower row, green fluorescence, red fluorescence, merge. *Scale-bar*: 10 μm

Individually caged flies that had been fed 1738G as tenerals were subsequently fed F1R1 on day 18. Samples of salivary exudate were examined on days 11–18 (flies infected with 1738G only) and then on days 29–42 (both 1738G and F1R1 potentially present); note that with regard to the development of F1R1, days 29–42 is equivalent to days 11–24. Trypanosomes (1738G) were present in the salivary probes of 12/32 (38 %) flies on day 11 and by day 14 had been detected in the majority of flies (26/32 = 81 %). The pattern of production of different developmental stages is shown in Fig. 3 and a typical sample is shown in Fig. 4. Production of asymmetric dividers, the migratory forms that initiate epimastigote infection in the SG, appeared to peak around 14 days post infected feed, although these forms continued to be recorded up to day 42 when the experiment terminated. Metacyclics, the mammalian infective stage, were first recorded at day 11 and were subsequently found in an increasing number of flies; of the flies recorded trypanosome positive by 18 days, 50 % (16/32) were found to have produced metacyclics. Thus, by day 18 when they received the second infective bloodmeal containing F1R1, at least half of the flies already had a mature SG infection of 1738G.

During the second stage of examining salivary probes from days 29–42, all developmental stages for 1738G continued to be recorded. Thus, a probe from an individual fly on a single day might contain a mixture of metacyclics, proventricular trypomastigotes and/or asymmetric dividers as shown in Fig. 4. This indicates that there is a steady stream of these early developmental stages through the foregut of the fly after the initial appearance. F1R1 developmental stages were also recorded in these salivary probes as shown in Fig. 4. The pattern of developmental stages recorded was more variable than for 1738G, but essentially similar with the first metacyclics recorded on day 30 (= day 12 with respect to development of F1R1). Thus both trypanosome clones, despite a difference of 18 days in the start of infection, had the same developmental stages passing through the foregut to the SG at the same time. However, 1738G had a longer time period overall in which trypanosomes could migrate to the SG, leading to its significantly higher rate of SG infection in GR [(21 + 63)/101 = 83 %] compared to ntGR [(28 + 9)/68 = 54 %) flies (Fisher’s exact test: *P* = 0.0001) (Table 3).

**Fig. 5:**
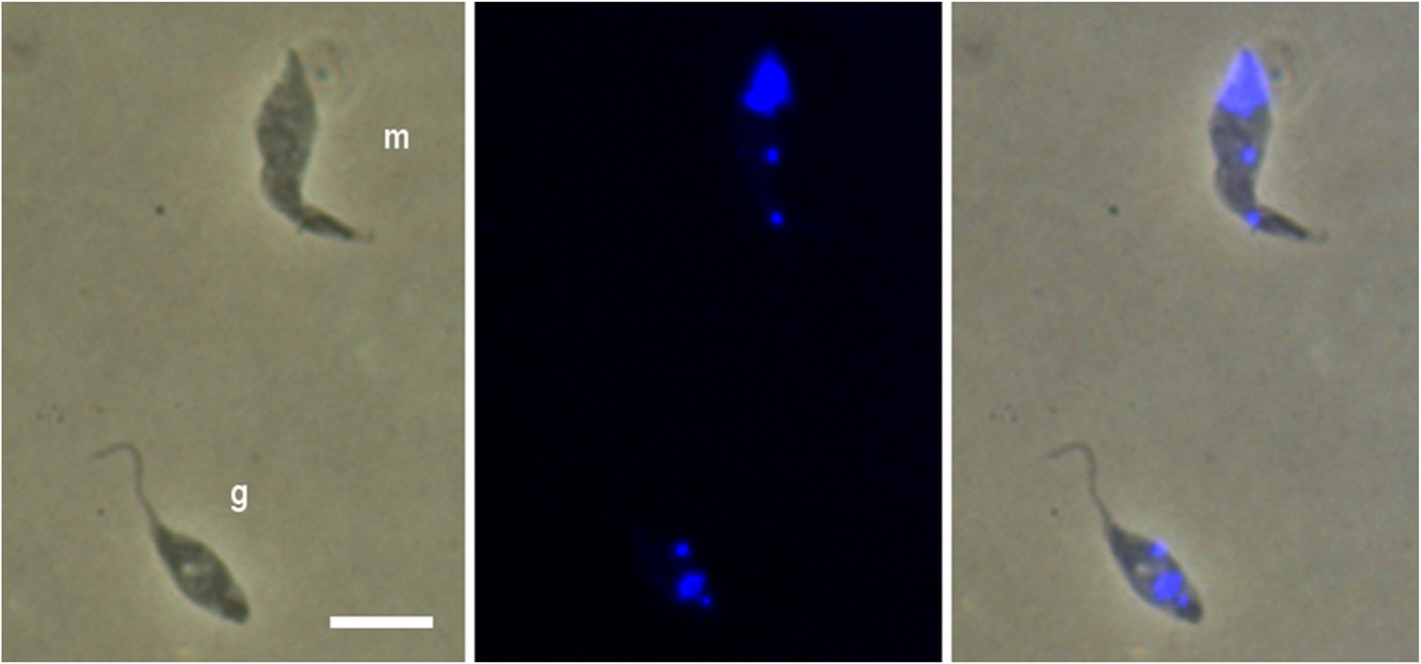
Sexual stages in salivary exudate. Trypanosomes in the salivary probe of a sequentially infected fly (*Glossina pallidipes*) 31 days after infection with 1738G and 13 days after infection with F1R1. Cells shown have the typical morphology of a haploid gamete (g) and a trypanosome in meiosis I (m). Both trypanosomes were red fluorescent, i.e. F1R1. From left to right, brightfield, DAPI and merge. *Scale-bar*: 5 μm

Previous experiments with a number of different *T. brucei* strains have demonstrated production of meiotic stages from days 14–38 post-infection [1, 2]. These stages were found attached inside the SG but were also recovered in the saliva from dissected SG. Similarly, the gamete stage was found in the saliva spilling from dissected SG [2]. For 1738, gametes were recorded from flies dissected on days 14–24 previously [2]. We therefore also examined the salivary probes for the presence of meiotic and gamete stages, as judged from their characteristic morphologies (Fig. 5). Table 5 shows that the timing of production of meiotic stages and gametes of 1738G and F1R1 overlapped during days 29–36, providing ample opportunity for mating in flies with both trypanosomes in the same SG.

**Table 5:** Evidence of meiotic stages and gametes in salivary probes. Percentage (and number) of flies (*Glossina pallidipes*) with meiotic or gamete stages in their salivary probes. 1738G was fed to teneral flies and F1R1 fed to these flies 18 days later; developmental day for F1R1 is given in brackets in first column

Developmental day 1738G (F1R1)	1738G meiotic	1738G gamete	FIR1 meiotic	FIR1 gamete
11	0	11 % (1/9)		
12	0	0		
13	17 % (1/6)	0		
14	0	0		
15	25 % (2/8)	50 % (4/8)		
16	60 % (3/5)	60 % (3/5)		
17	56 % (5/9)	56 % (5/9)		
18	17 % (2/12)	17 % (2/12)		
29 (11)	0	33 % (3/9)	11 % (1/9)	11 % (1/9)
30 (12)	0	38 % (3/8)	0	13 % (1/8)
31 (13)	23 % (3/13)	38 % (5/13)	15 % (2/13)	23 % (3/13)
32 (14)	25 % (1/4)	0	0	0
33 (15)	44 % (4/9)	33 % (3/9)	11 % (1/9)	22 % (2/9)
34 (16)	0	50 % (2/4)	25 % (1/4)	25 % (1/4)
35 (17)	27 % (3/11)	27 % (3/11)	0	9 % (1/11)
36 (18)	0	38 % (3/8)	0	13 % (1/8)
38 (20)	45 % (5/11)	18 % (2/11)	0	0
40 (22)	27 % (3/11)	27 % (3/11)	0	0
42 (24)	17 % (2/12)	8 % (1/12)	0	0

## Discussion

In *T. brucei* sexual reproduction with production of hybrid genotypes occurs when there is a mixture of different trypanosome strains in the tsetse salivary glands. As tsetse become refractory to infection after their first bloodmeal [3–8], it was assumed that a mixed infection would most likely be acquired on the first feed, for example from an animal with a mixed infection. This would restrict opportunities for trypanosomes to mate in nature; for example, humans are infected with *T. b. rhodesiense* (*Tbr*) not *T. b. brucei* (*Tbb*), so in order to acquire a mixed infection of both subspecies, a fly would need to feed on a non-human reservoir host of *Tbr* also infected with *Tbb*.

On the other hand, it has been shown that starvation increases the chance that mature tsetse flies acquire trypanosome infection [16, 18], and starvation is a condition that tsetse flies commonly encounter in nature when host animals are scarce. Therefore, we hypothesized that starved flies might offer a suitable environment for the development of mixed trypanosome infections and production of trypanosome hybrids. Although starved 18 day old flies had reduced infection rates compared to flies infected with trypanosomes as tenerals, they still developed a high proportion of mixed infections and produced hybrids.

In flies sequentially infected with different trypanosome clones 18 days apart, a well-established trypanosome infection from the first bloodmeal severely reduced the ability of the second incoming trypanosome strain on day 18 to colonise the midgut. This effect was additional to the age-related refractory nature of 18 day old flies, and appears to be the result of a specific immune response to the pre-existing trypanosome infection. Considering recently proposed models of tsetse immunity [8, 10], the dense population of trypanosomes within the ectoperitrophic space in an 18-day old infection has been in direct contact with the immune-reactive midgut epithelium for some time (~11 days), so that presumably the second incoming trypanosome strain confronts very high levels of immune effectors in the midgut. Previous analyses of the activity of trypanocidal factors in the tsetse midgut such as attacin [32], peptidoglycan recognition protein (PGRP-LB) [33, 34], reactive oxygen intermediates (ROI) [14, 27] and tsetse EP protein [35, 36] have tended to focus on trypanosome-challenged flies rather than those with an established infection, but there are some results for older flies. Increased expression of attacin was observed in the proventriculus and fat body of 20-day-old *T. brucei* infected flies, but there was no change in activity of nitric oxide synthase, nitric oxide or hydrogen peroxide compared to controls [14]. In non-teneral flies, RNA knockdown of either tsetse EP protein or Relish, the transcriptional activator of antimicrobial peptide (AMP) genes via the immunodeficiency (IMD) pathway, led to increased midgut infection rates with *T. brucei* [13, 36], suggesting that tsetse EP protein and/or AMPs could be key players in the immunity to sequential infection observed here. However, starvation for 3 days or more noticeably reduces the level of tsetse EP protein [36], downplaying its potential role here. We also used the antioxidant glutathione to increase midgut infection rates in the sequentially fed flies, thereby eliminating the possibility that ROI play a major role in immunity to the second fed trypanosome. The upregulation of immune responses appears to be restricted to the midgut of flies with an established infection, as SG invasion and colonisation was unaffected in sequentially fed flies, which had comparable transmission indices to teneral flies.

Although relatively few flies developed a mixed infection of the midgut after sequential feeding, some also developed a mixed SG infection and hybrids were produced. This contrasts with the results of an analogous study on the mosquito-*Plasmodium* system, where a pre-existing *Plasmodium chabaudi* infection in the *Anopheles stephensi* vector enhanced the establishment of a second *P. chabaudi* strain; however, there is no advantage for *P. chabaudi* in terms of increased opportunity to mate, because production of gametes from the two infections is asynchronous [19]. In trypanosomes meiotic dividers and gametes were produced continuously, providing the opportunity for gametes from early and late infections 18 days apart to coincide in the SG, resulting in hybrids. Moreover, SG infection is not the result of a single episode of invasion and colonisation, but a continuous process.

It is therefore plausible that in nature *T. brucei* strains can establish co-infection in the vector and undergo mating after being picked up sequentially by hungry flies feeding on different hosts. This has implications for the frequency of mating between *Tbr* and *Tbb*, since humans cannot carry a mixed infection of both subspecies. We previously showed that new strains of *Tbr* were generated in *Tbr*/*Tbb* crosses when the serum resistance associated (*SRA*) gene from *Tbr*, which confers human infectivity [37], was transferred into a new genetic background [38]. Even if rare, such events are of epidemiological significance in that new strains of the pathogen are generated that have combinations of genes not previously encountered by the human population. In models of the epidemiology of African human trypanosomiasis, feeds from animals are sometimes regarded as “empty feeds” that dilute the probability of human infection [39]. Instead it can be seen that flies with heterogeneous host feeding preference that mostly feed on livestock with occasional feeds on humans may likely acquire *Tbb* and *Tbr* sequentially, thus enabling the potentially dangerous transfer of virulence factors to create new pathogen strains.

## Conclusion

We found that a second trypanosome strain can establish infection in the tsetse salivary glands 18 days after the first infected feed, with co-mingling of gametes and production of trypanosome hybrids. Establishment of the second strain was severely compromised by the strong immune response of the fly to the existing infection. Although sequential infection provides an opportunity for trypanosome mating, the easiest way for a tsetse fly to acquire a mixed infection is by feeding on a co-infected host.

## Abbreviations

AMP, antimicrobial peptide; CM, Cunningham’s medium; GFP, green fluorescent protein; IMD, immunodeficiency; PBS, phosphate buffered saline; PFA, paraformaldehyde; RFP, red fluorescent protein; ROI, reactive oxygen intermediate; SG, Salivary glands; *Tbb*, *Trypanosoma brucei brucei*; *Tbr*, *Trypanosoma brucei rhodesiense*; TI, transmission index
